# Impact of the COVID-19 pandemic on the mental health of hospital employees: single center experience

**DOI:** 10.1192/j.eurpsy.2024.766

**Published:** 2024-08-27

**Authors:** M. Kristic, V. Kuresic, I. Miskulin, J. Dumic, L. Dumic, M. Miskulin

**Affiliations:** ^1^Department of Public Health, Faculty of Medicine Osijek; ^2^Department of Dental Medicine, Faculty of Dental Medicine and Health Osijek, Osijek, Croatia

## Abstract

**Introduction:**

Hospital employees are at high risk of developing mental health issues during the coronavirus (COVID-19) pandemic. Indeed, several studies have shown increased rates of anxiety, depression, stress, and other mental health issues but existing studies show inconsistencies, and each country has some local specificities.

**Objectives:**

This study aimed to investigate the influence of the COVID-19 pandemic on various aspects of the mental health of hospital employees (health workers and non-health workers) from Croatia.

**Methods:**

This cross-sectional questionnaire study was conducted from February to April 2023 period. A validated, anonymous questionnaire that contained questions regarding demographic data, as well as the Pittsburgh Sleep Quality Index (PSQI), the Zung Self-Rating Anxiety Scale, and the Zung Self-Rating Depression Scale was self-administered to a convenient sample of hospital employees from one general hospital in northwestern Croatia.

**Results:**

The study sample included 360 subjects with a median age of 42 years (interquartile range 35-50), 24.7% males, and 75.3% females. According to the PSQI, 21.1% of subjects presented sleep disturbances. According to the Zung Self-Rating Anxiety Scale, there were 39.4% of subjects with anxiety while according to the Zung Self-Rating Depression Scale, there were 6.4% of subjects with depression. Sleep disturbances were more frequent among subjects who considered their socioeconomic status as under average (p=0.040), and among health workers in comparison to non-health workers employed in hospital (p=0.040). Anxiety was more frequent among females (p=0.010), and subjects with lower levels of education (only elementary school) (p=0.040). Depression was more frequent among females (p=0.030).

**Conclusions:**

The COVID-19 pandemic has a significant negative influence on the mental health of hospital employees where health workers in comparison to non-health workers, females, subjects with lower levels of education, and subjects who considered their socioeconomic status as under average are more prone to the development of investigated mental health issues. The development of appropriate supportive programs that enhance the mental health of all hospital employees during pandemics is needed to address mental health issues in this vulnerable population.

**Disclosure of Interest:**

None Declared

